# Biocontrol Potential of Native *Trichoderma* Strains Toward Soil-Borne Phytopathogenic and Saprotrophic Fungi

**DOI:** 10.3390/jof11070535

**Published:** 2025-07-18

**Authors:** Kristina Atlagić, Tijana Cvetić Antić, Jovana Lukičić, Katarina Kruščić, Miroslav Živić, Nikola Unković, Tanja Pajić, Katarina Stevanović, Nataša V. Todorović

**Affiliations:** 1 University of Belgrade—Faculty of Biology, Studentski trg 16, 11158 Belgrade, Serbia; kristina.tesanovic@bio.bg.ac.rs (K.A.); tcvetic@bio.bg.ac.rs (T.C.A.); jovana.lukicic@bio.bg.ac.rs (J.L.); katarina.kruscic@bio.bg.ac.rs (K.K.); mzivic@bio.bg.ac.rs (M.Ž.); unkovicn@bio.bg.ac.rs (N.U.); 2 Institute for Biological Research “Siniša Stanković”, National Institute of the Republic of Serbia, University of Belgrade, Bulevar Despota Stefana 142, 11000 Belgrade, Serbia

**Keywords:** biocontrol, *Trichoderma*, peptaibol, *Sclerotinia sclerotiorum*, *Rhizoctonia solani*, *Phycomyces blakesleeanus*, alamethicin, antioxidative defense, growth inhibition

## Abstract

The potential of *Trichoderma* fungi as biocontrol agents has not yet been fully explored, as there is a large repertoire of inter- and intra-species variation in their phytopathogenic antagonistic effects due to different adaptations of individual *Trichoderma* strains. In the present study, we investigated the biocontrol efficacy of eight native isolates of *Trichoderma* spp. against the soilborne phytopathogens *Sclerotinia sclerotiorum* and *Rhizoctonia solani* and a representative of the Mucoromycota, *Phycomyces blakesleeanus*. An *in vitro* dual culture test showed a complete (100%) inhibition of *S. sclerotiorum* and *P. blakesleeanus* by each tested *Trichoderma* strain and a high (80–100%) inhibition of *R. solani*. The crude chloroform extracts, whose peptide contents were confirmed by thin-layer chromatography, caused a concentration-dependent reduction in the growth of the target fungi, with inhibition comparable to the effect of the peptaibol standard alamethicin. Despite the differences between fungi from the phyla Basidiomycota, Ascomycota, and Mucoromycota, their inhibition by alamethicin followed the same dose–response dependence. The growth inhibition of *P. blakesleeanus* induced by *Trichoderma* extracts was characterized by a significantly increased activity of antioxidative defense enzymes. Both variants of biocontrol agents, the native strains of *Trichoderma* spp. and their extracts, are efficient in controlling fungal growth and should be considered for the development of new potent bioformulations applicable in agriculture.

## 1. Introduction

In recent decades, crop yields have been increasing thanks to intensive farming and the widespread use of pesticides, which contribute significantly to food security [1]. However, climate change, including warming, extreme weather events, and severe droughts, is predicted to lead to significant crop losses in the future [2]. In addition to the well-documented increasing threat of widespread resistance to nearly all groups of currently used chemical fungicides [3], the systematic use of chemical fungicides also has a negative impact on the environment, as it affects non-target organisms, including beneficial soil microbes [4], and has a negative impact on the population balance of arthropods [5]. Alternative approaches already exist thanks to the development of alternative “green” biopesticides, some of which are bacteria-based [6], while others are formulated as fungus-based agents. *Trichoderma* is a genus of fungi known for its role as biocontrol agents in agriculture and is responsible for about 50–60% of the biofungicides available worldwide [7]. The complexity of their ecophysiology and the diversity of *Trichoderma* spp. highlight their potential as direct and indirect biocontrol agents, biostimulants, and biofertilizers with diverse potential applications in agriculture [8].

*Trichoderma* fungi suppress phytopathogens through various mechanisms such as competition, antibiosis, and mycoparasitism [7,9]. Characteristically, they produce a variety of secondary metabolites (SMs) that have beneficial effects on plants, promoting their growth and boosting their intrinsic antimicrobial defenses [10], while others are various potent antimicrobial compounds [11]. SMs can perform various activities, such as antifungal, antibacterial, antitumor, antioxidant, and anti-inflammatory effects [12]. Peptaibols, a specific class of SM released by *Trichoderma* species, are non-ribosomally synthesized short peptides characterized by the presence of α-aminoisobutyric acid (Aib), often terminating in an alcohol group, and play an important role in the antifungal and antimicrobial activities of *Trichoderma* species [13]. Peptaibols act in synergy with other secondary metabolites and cell wall-degrading enzymes [14], leading to cell lysis and death of the pathogen [15]. The various chitinase enzymes, which act in combination with a number of glucosanases and proteases [14], degrade the cell wall of target fungi [16]. In higher fungi, chitin accounts for up to 45% of the cell wall, whereas the dominant cell wall component of pathogens from the phylum Mucoromycota is chitosan, which accounts for up to 40% of the mass, while chitin contributes only 20%, with a minimal contribution from β-glucans bound to a specific chitin subtype [17,18]. The different composition of the cell wall of fungi belonging to the phylum Mucoromycota compared to fungi belonging to the Ascomycota and Basidiomycota might prevent one of the steps in the *Trichoderma* mycoparasitism cascade and thus provide immunity against attacks by *Trichoderma* species. Since some members of the Mucoromycota are plant pathogens, such as *Choanephora cucurbitarum* [19,20] and *Choanephora infundibulifera*, *Mucor racemosus*, and *Rhizopus stolonifer* [21], it would be useful to include Mucoromycota in the evaluation of antagonistic activity of new isolates or future *Trichoderma*-based biocontrol agents. Various *Trichoderma* species have been studied for their antagonistic activity against *Rhizoctonia solani* [22,23], an important soil-borne pathogen of numerous economically important plant crops and useful plants with worldwide distribution [24]. The mechanisms and efficacy of *Trichoderma* antagonism against *R. solani* appear to be species- and strain-dependent [25]. Several studies showed that *Trichoderma* spp. express genes encoding proteases and oligopeptide transporters before and during interactions with *R. solani* [26]. Sensing of the presence of *R. solani* through the detection of released peptides and other small molecules, such as reactive oxygen species (ROS), triggers signaling cascades that enable an appropriate biochemical response of *Trichoderma* spp., leading to appressoria formation, coiling, and mycoparasitism [27]. This is followed by the subsequent activation of production and release of secondary metabolites, such as small, non-ribosomally synthesized peptides peptaibols [28], diketopiperazines, including epipolythiodioxopiperazines (such as gliotoxin and gliovirin) [29], ribosomally synthesized and post-translationally modified peptides [30], cell wall-degrading enzymes, and other small molecules (polyketides, isoprenoid-derived metabolites, pyrones, etc.) with antifungal activity that ultimately kill the phytopathogen [7]. Similar cascades of events have been described in the interaction of different *Trichoderma* species and strains with *Sclerotinia sclerotiorum* [31].

To date, broad-spectrum chemical pesticides have often proven to be more efficient than commercially available *Trichoderma*-based biocontrol agents [32]. Because of the pressing need for the development of more efficient and sustainable biocontrol formulations [33], and in response to the urgent calls for a complete reorganization of agricultural practices [34] according to the integrated pest management paradigm [35], new *Trichoderma* strains need to be researched and evaluated for their efficacy against the major phytopathogenic fungi.

The present study investigates the antifungal activity of eight native *Trichoderma* isolates against two native phytopathogens, *Rhizoctonia solani* (Basidiomycota) and *Sclerotinia sclerotiorum* (Ascomycota), which are known to contribute to massive crop losses, and a model fungus, *Phycomyces blakesleeanus* (Mucoromycota). *Sclerotinia sclerotiorum* is a plant pathogen of the aerial parts of plants and roots that causes white mold, a disease that can develop during the growing season and in the post-harvest period and is associated with significant losses in a variety of affected plant hosts worldwide (soybeans, green beans, lettuce, carrots, etc.). *Rhizoctonia solani* is a soil-borne plant pathogen that attacks a variety of host plants (corn, rice, wheat, barley, oats, soybeans, peanuts, potatoes, sugar beets, coffee, etc.) and is ubiquitously distributed. *Phycomyces blakesleeanus*, a well-established model organism, belongs to a diverse monophyletic group that includes numerous species of plant pathogens, opportunistic pathogens, and saprotrophs [36]. It has been used in recent studies on patch-clamp registration of fungi ion currents through the plasma membrane from sporangiophores [37,38] and hypha protoplasts [39], as well as antioxidative defense enzyme activity changes and biotransformation ability [40,41], and has been studied in detail by the Third Harmonic Generation imaging technique, which is particularly applicable for lipid droplets [42]. However, the susceptibility of *P. blakesleeanus* toward *Trichoderma* isolates has not been investigated up to now, to the best of our knowledge.

The antagonistic potential of *Trichoderma* spp. isolates against the target fungi was measured in direct contact *in vitro* by the dual culture method, while the inhibitory effect of crude extracts containing SMs released by *Trichoderma* was tested using the well-dilution assay and compared with the effect of the peptaibol standard alamethicin. Furthermore, it was shown that the extracts obtained from *Trichoderma* contain peptaibol-like components and that they induce an increase in the activity of oxidative stress defense enzymes in the treated fungi. All tested *Trichoderma* strains from Serbia proved to be effective biocontrol agents against the tested fungi and are promising candidates for the development of new biofungicidal formulations that will help to minimize the use of chemical control measures.

## 2. Materials and Methods

### 2.1. Fungal Strains and Cultivation Conditions

Eight native strains of *Trichoderma* species, *T. citrinoviride*—NK 1/9, 127, and 11H1-4, *T. harzianum*—M5/1, 1H1-27 and 1S2-6, *T. afroharzianum*—1S2-8, and *T. longibrachiatum*—2S2-31, from the fungal culture collection of the Faculty of Biology, University of Belgrade, were cultivated. *Trichoderma harzianum*—1H1-27 and 1S2-6, *T. citrinoviridae*—11H1-4, *T. afroharzianum*—1S2-8, and *T. longibrachiatum*—2S2-31 were soil-borne fungi isolated from the river flow of the Danube River (Belgrade, Serbia). Strain *T. citrinoviride*—NK 1/9 was an air-borne fungus obtained from indoor ambient conditions in Gadžin Han, Serbia; strain *T. harzianum*—M5/1 originated from deteriorated wood located in Kostolac, Serbia. The isolation of strain *T. citrinoviride*—127 from the air of indoor ambient conditions in Belgrade, Serbia, was already published [43], but its activity has not been investigated. Two phytopathogens indigenous in the fields of the Vojvodina region, *Sclerotinia sclerotiorum*—K500 and *Rhizoctonia solani*—K499, were obtained from the collection of phytopathogenic fungi of the Institute of Field and Vegetable Crops, Novi Sad, Serbia. All the abovementioned isolates were cultured for 7 days at 25 ± 2 °C on potato dextrose agar (PDA) (Titan Biotech, Delhi, India). *Phycomyces blakesleeanus*, wild-type strain NRRL 1555 (−), was cultured for 7 days on PDA in growth boxes with continuous overhead white light at 20 ± 2 °C and approximately 95% relative humidity [44].

### 2.2. Molecular Identification of the Fungi

Fungal mycelia (~100 mg), pre-crushed with liquid nitrogen, were harvested for total genomic DNA extraction using the ZR Fungal/Bacterial DNA Mini Prep KIT according to the manufacturer’s protocol (Zymo Research, Irvine, CA, USA). One gene region, ITS II (internal transcribed spacer 1, partial sequence; 5.8S ribosomal RNA gene and internal transcribed spacer 2, complete sequence; and large subunit ribosomal RNA gene), was amplified, and additional *tef1* regions were used to more reliably identify *Trichoderma* [45,46]. The primers used to amplify the above regions and the corresponding PCR profiles are listed in Table S1 in the Supplementary Material.

PCR was performed in a 25 µL final volume, as described in Six et al. [47] (2011). Each PCR reaction mixture (25 µL total volume) consisted of 10.5 µL ultra-pure DNase/RNase-free water (Gibco, Leicestershire, UK), 12.5 µL Fast Gene Taq ready mix with dye, 250 × 50 µL, 0.5 µL of each primer (10 µM), and 1 µL of DNA extract. The PCR conditions were one cycle of denaturation at 95 °C for 4 min, followed by 35 cycles of denaturation at 95 °C for 30 s, annealing at 52 °C for 60 s, extension at 72 °C for 60 s, and a final cycle of extension at 72 °C for 10 min. The PCR products were purified using the EXTRACTME DNA CLEAN-UP KIT according to the manufacturer’s protocol (BLIRT, Gdańsk, Poland). The purified PCR products were sequenced as a commercial service by Eurofins Scientific (Augsburg, Germany). The edited sequences were aligned together with the sequences of reference strains from the GenBank database, using the ClustalW algorithm within BioEdit software (version 7.2.5), to assess sequence similarity.

A phylogenetic tree (shown in Supplementary Material, Figure S1) was constructed in MEGA 11.0.13 software by the neighbor-joining method based on a pairwise distance matrix, obtained with the Kimura two-parameter nucleotide substitution model, and evaluated using the bootstrap resampling method with 1000 replicates.

### 2.3. Confrontation Assay

To evaluate the antagonistic effect of the investigated *Trichoderma* strains on *S. sclerotiorum* and *R. solani*, as well as *P. blakesleeanus*, the dual culture method was used. *Trichoderma* strains and phytopathogens were cultured individually on PDA for 5 days at 26 ± 2 °C. Mycelial plugs (5 mm diameter) of *Trichoderma* spp. and phytopathogens were cut and inoculated into the same Petri dish with PDA at defined positions 3 cm apart. The plates were incubated for 7 days in a thermostat (UE 500, Memmert, Schwabach, Germany) at 26 ± 2 °C in the dark. For dual cultures of *Trichoderma* strains with *P. blakesleeanus*, the fungi were cultured at ambient temperature (20 ± 2 °C) and exposed to light on PDA media enriched with thiamine (1 mg/L) and yeast extract (1.5 g/L). Plugs from *P. blackesleeanus* mycelium were sampled from monocultures for dual culture initiation after two days of growth to avoid the formation of giant sporangiophores. The monocultures of the individual fungus were grown at the same time under the same conditions as the control cultures. The antagonistic effects of *Trichoderma* were documented on the seventh day of incubation by measuring colony radius lengths and photographing all confrontation culture plates using a standardized procedure. The experiments were carried out in triplicate. Growth inhibition was quantified using the Radial Growth Inhibition (RGI) method and the Bio-Control Index (BCI) method. The radial growth inhibition index was calculated using the following formula: RGI (%) = [(r1 − r2)/r1] × 100, where r1 = radius of the control colony (in mm) and r2 = radius (in mm) of the colony confronted with a *Trichoderma* isolate [48]. For BCI measurements, photographs of each plate were taken with a Canon EOS 4000D digital camera (Tokyo, Japan) mounted on a specially designed tripod at the same distance from all plates, placed in the same position, with a plate holder. The visible area of the *Trichoderma* colony and the total area occupied by both the *Trichoderma* colonies and the target fungal colony were measured using the freehand selection tool in ImageJ2 software (ImageJ 2.9.0/1.54p, W. Rasband, National Institute of Health, Bethesda, MD, USA). The images were spatially scaled, and the areas of the colonies were outlined and measured (Analyze > Measure). The BCI was calculated according to the formula *BCI* = *T*/(*T* + *P*) × 100, where *T* is the area of the *Trichoderma* colony and *T* + *P* is the total area occupied by the colonies of both *Trichoderma* and the target fungus [49].

### 2.4. Isolation of Crude Extracts

Crude extracts of *Trichoderma* cultures were prepared according to the modified procedure of Tamandegani et al. [50]. The plates from the confrontation assay were used for isolation. The surface of each plate (containing the axenic (pure) *Trichoderma* culture or the dual culture of *Trichoderma* and plant pathogen), prepared in the same way as for the confrontation test, was flooded twice with 5 mL of chloroform (Fisher, Leicestershire, UK). The mixture of chloroform and *Trichoderma* mycelia was then collected and shaken for 2 h at room temperature (Orbital Shaker Grant-bio PSU-20i, Grant Instruments Ltd., Royston, UK). The chloroform was then evaporated to dryness overnight in a fume hood. The dry residues were dissolved in 1.5 mL of methanol (Fisher, UK) per plate. The resulting crude extracts were centrifuged for 10 min at 10,000 rpm (Tehtnica, Centric 200R, Železniki, Slovenia) to remove chloroform-resistant mycelial residues. The supernatant was transferred to pre-weighed tubes and left in the fume hood overnight. After evaporation of the methanol, the mass of the dry residue was measured, and the dry residues were resuspended in 200 µL of methanol and stored at −20 °C for further analysis.

### 2.5. Thin-Layer Chromatography (TLC) Analysis

Crude peptaibol-containing extracts dissolved in methanol (8 µL per spot) were applied to pre-coated TLC silica gel 60 F254 plates (Merck, Darmstadt, Germany) and developed in a solvent mixture, i.e., CHCl_3_:MeOH (8:2), in a pre-equilibrated chamber. Alamethicin (Santa Cruz Biotechnology, Dallas, TX, USA), 4 µL of a 2.5 mg/mL stock solution in methanol, was used as a standard. Peptaibol bands were detected by spraying with HCl, followed by spraying with ninhydrin (0.5% *w*/*v* in 1-butanol), according to the method of Pandey et al. [51].

### 2.6. Well Diffusion Method

The antifungal effect of the crude extracts of eight *Trichoderma* isolates was tested by the well diffusion method in serial microdilutions on cultures of *S. sclerotiorum*, *R. solani*, and *P. blakesleeanus*. Agar plates with 20 mL of a medium corresponding to the tested culture (PDA for *S. sclerotiorum* and *R. solani* and PDA with yeast extract and vitamin B for *P. blakesleeanus*) were modified as follows: For crude extract testing, five equidistant round wells of 5 mm diameter were bored into the agar plates on the periphery of the Petri dish at equal distance from the center of the plate and filled with 25 µL of serial dilution of a crude *Trichoderma* extract in methanol (100%, 50%, 25%, 12.5%). Methanol was used in one well as a solvent control. Subsequently, the mycelial plugs of phytopathogens and *P. blakesleeanus* were placed in the center of the plate, and cultures were grown under standard growth conditions for the respective fungus for 7 (*S. sclerotiorum*, *R. solani*) and 6–7 days (*P. blakesleeanus*), respectively. The diameter of the growth inhibition zones was measured at the end of the growth period. Alamethicin, a commercially available linear peptaibol, was tested against selected fungal species *S. sclerotiorum*, *R. solani*, and *P. blakesleeanus* as a peptaibol standard, at the same time and under the same conditions, with extract well diffusion plates as follows. Eight equidistant round wells with a diameter of 5 mm were drilled into the agar plates on the periphery of the Petri dish, and 25 µL of alamethicin at successive dilutions (in µg/mL) (2500, 1250, 625, 312.50, 156.25, 78.25, and 25) was added to the wells. The control well on each plate was filled with 25 µL of methanol.

### 2.7. Calculation of the Dry Substance Concentration of the Crude Extract That Causes a Minimum Observable Inhibition of Mycelial Growth (MIMGI) of the Target Fungi

For each combination of a crude extract obtained from a *Trichoderma* strain with a plant pathogen in the well diffusion assay, the minimum extract dilution causing growth inhibition of the target fungal colony was determined. The minimal concentration inducing inhibition of mycelia growth (MIMGI) value, which corresponds to the content of the dry substance in the effective dilution of the extract, was calculated by multiplying dilution coefficient with the mass of the dry chloroform extract measured for each combination of *Trichoderma* strain with the plant pathogen during the described preparation of the crude extract.

### 2.8. Treatment of the Liquid Culture of P. blaeksleeanus with Crude Extract and Preparation of the Mycelial Lysate for the Measurement of Enzyme Activity

The spores of the wild type of *P. blakesleeanus* (NRRL 1555(−)) (10^7^) were activated by temperature (15 min in a water bath at 49.5 °C) and grown in 20 mL of standard minimal medium (SMM) in shaken Erlenmeyer flasks in a growth cabinet with continuous overhead white fluorescent light of 10 Wm^−2^ at a temperature of 21 °C [52]. The 20 h old mycelium was drained under vacuum and treated with 3.5 mL of a 12.5% crude extract diluted in SMM for 90 min. Only *T. harzianum*-1S2-6 extract was tested. The mycelial samples were then replenished with fresh SMM to a total volume of 20 mL and shaken until they were 44 h old from the start. Vacuum-drained mycelia samples were weighed and frozen in liquid nitrogen for further analysis.

The frozen mycelium samples were homogenized in liquid nitrogen using a mortar and pestle and suspended in 100 mM potassium phosphate buffer, pH 7.2, with 2 mM ethylenediaminetetraacetic acid (EDTA) and 0.5 mM phenylmethylsulfonyl fluoride. The ratio of mycelium to buffer was 1:5. After 30 min of extraction on ice, the homogenate was centrifuged (10 min at 10,000× *g*), and the supernatant was used as protein extract.

### 2.9. Quantification of the Protein Content and Enzyme Activities in the Mycelial Lysate

The protein content was estimated according to Bradford [53], modified for a microtiter plate reader, with bovine serum albumin (BSA) as the standard protein. All enzyme assays were performed at 22 °C. Catalase (CAT) was assayed polarographically using a Clark-type liquid-phase oxygen electrode (Hansatech Oxygraph, Norfolk, UK) following the method of Del Rio et al. [54]. The reaction was started by adding 2 µL of mycelial lysate to the reaction chamber containing 100 mM potassium phosphate buffer and 12 mM H_2_O_2_, in a total volume of 1 mL.

Glutathione peroxidase (GPx) was assayed spectrophotometrically by monitoring the depletion of nicotinamide adenine dinucleotide phosphate (NADPH). The reaction mixture contained 2 mM glutathione (GSH), 200 µM NADPH, 2 mM cumene hydroperoxide, 1 mM EDTA, and 2.5 U glutathione reductase (GR) in 100 mM potassium phosphate buffer, pH 7 [55], in a total volume of 1 mL. The reaction was started by adding 20 µL of mycelial lysate.

The glutathione S-transferase activity was determined spectrophotometrically according to the method of Habig et al. [56]. The reaction mixture (1 mL) contained 1 mM GSH and 1 mM 1-chloro-2,4-dinitrobenzene (CDNB) in 100 mM potassium phosphate buffer, pH 6.5, and the reaction was started by adding 20 µL mycelial lysate.

### 2.10. Statistical Comparisons and Curve Fitting

GraphPad Prism 9.0 software was used for statistical comparisons, curve fitting, and graph generation. For fitting the dose–response to alamethicin, the GraphPad sigmoid 4pl function was used, with a variable slope for X as concentration: $Y=Bottom+(X^{n_{h}})*(Top-Bottom)/(X^{n_{h}}+{{IC}_{50}}^{n_{h}})$. In the equation, Y is the response (measured diameter of the inhibition zone, in mm); Bottom and Top are the minimum and maximum values of the response; IC_50_ is the half maximum concentration, corresponding to the concentration of alamethicin that achieves the 50% of the maximum inhibition; and n_h_ is the slope of the sigmoid curve, i.e., the Hill slope. Fitting was performed by taking into account all values in the graph, not just the mean values, with a 95% confidence level. The confidence intervals (CIs) for the parameters were set to be symmetrical asymptotic, in order to obtain the standard error (SE) of the fit parameters. To test whether two data sets can be fitted to the same curve, the extra sum-of-squares F test was used, with *p* < 0.05 as the cut-off value for selecting the simpler model.

For statistical comparisons of two or more groups of data, two-way or one-way ANOVA, mixed and simple models, without repetition, were used, always with the Holm–Sidac correction for multiple comparisons. The confidence levels for statistical significance were 0.05 (*), 0.01 (**), 0.005 (***), and 0.0001 (****). All results are reported as mean ± SE, unless otherwise stated.

## 3. Results

### 3.1. Sequence Analysis

Molecular identification of eight *Trichoderma* isolates obtained from two culture collections in Serbia indicated the presence of a total of four species: *T. afroharzianum*, *T. citrinoviridae*, *T. harzianum*, and *T. longibrachiatum*. The pathogenic fungi were identified as *S. sclerotiorum* and *R. solani*. All identified fungi used in this study, with strain codes and accession numbers, are summarized in Table 1.

### 3.2. Susceptibility of the Tested Fungi to Trichoderma Isolates

**Figure 1:**
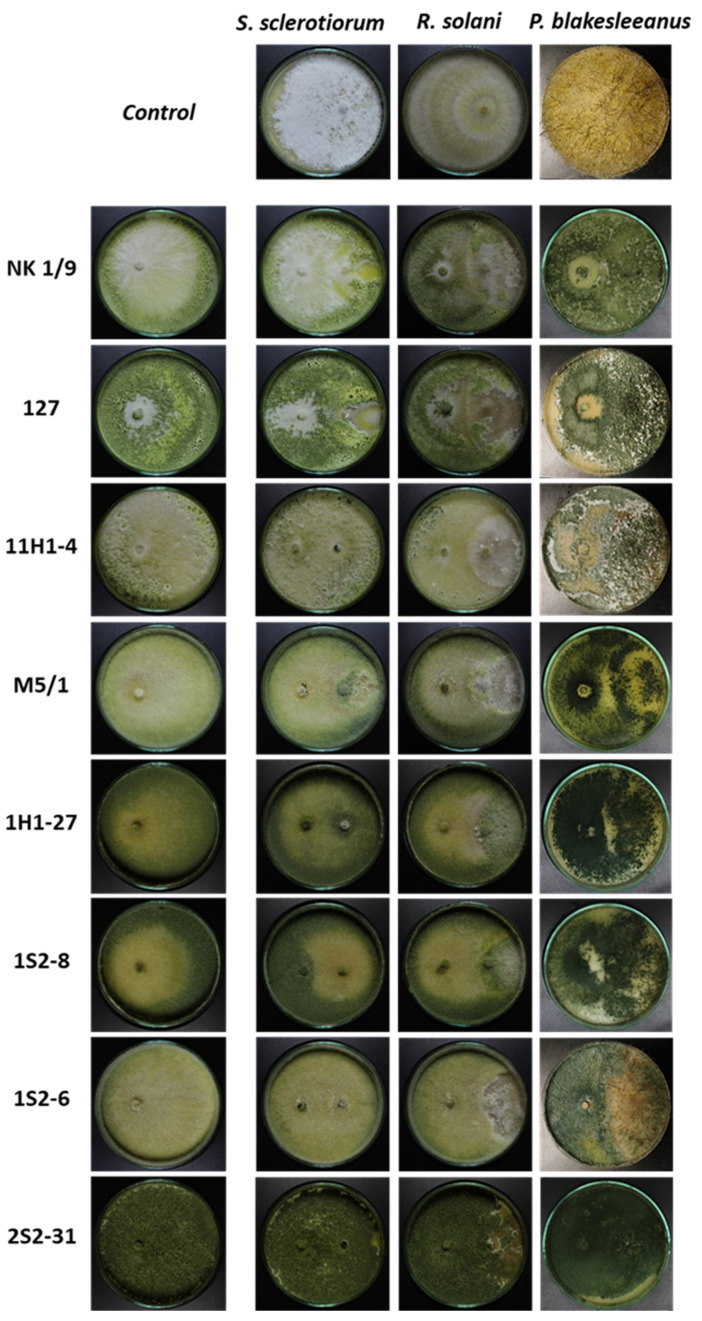
Representative images of Petri plates showing the results of confrontation (7 days of incubation) of *Trichoderma* spp. strains with each target fungus tested. From top to bottom, strains of *Trichoderma* spp.: NK 1/9, 127, 11H1-4, M5/1, 1H1-27, 1S2-8, 1S2-6, and 2S2-31. All experiments were performed in triplicate. The images on the far left show *Trichoderma* spp. control cultures obtained in phytopathogen growth conditions. Control cultures corresponding to *P. blakesleeanus* confrontation plates are shown in Figure S3.

**Figure 2:**
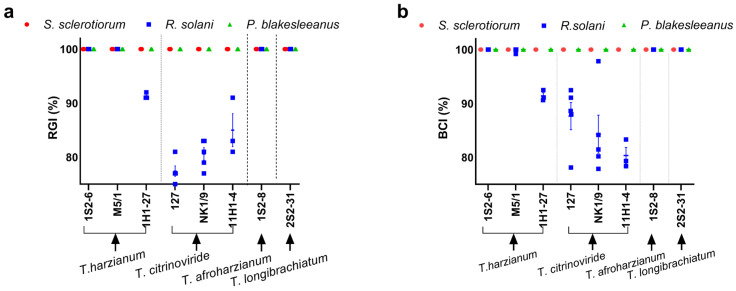
Confrontation (after 7 days of incubation) essay quantification results. Dual cultures of individual *Trichoderma* spp. isolates (NK 1/9, 127, 11H1-4, M5/1, 11H1-27, 1S2-8, 1S2-6, 2S2-31) against *S. sclerotiorum*, *R. solani*, and *P. blakesleeanus*. (**a**) Antagonistic activity of each *Trichoderma* spp. strain quantified by the RGI method. (**b**) Antagonistic activity of each *Trichoderma* spp. strain, quantified by the BCI method. Mean ± SE (*n* = 3), all points shown. *S. sclerotiorum*: red circles; *R. solani:* blue squares; *P. blakesleeanus*: green triangles.

The antagonistic activity of *Trichoderma* spp. against *S. sclerotiorum*, *R. solani*, and *P. blakesleeanus* was determined by confrontation tests in dual cultures after 7 days of incubation (Figure 1). Both the RGI and the BCI methods yielded comparable results (Figure 2a,b). The RGI obtained by confronting *Trichoderma* isolates with the tested phytopathogens was between 78 and 100%, while the BCI was between 80 and 100%. The greatest reduction in the growth of *S. sclerotiorum* and *R. solani* (100%) was observed when confronted with *T. afroharzianum*—1S2-8, *T. harzianum*—1S2-6 and M5/1, and *T. longibrachiatum*—2S2-31. Another isolate of *T. harzianum*—1H1-27 showed the maximum measurable antifungal activity against *S. sclerotiorum* (RGI and BCI 100%), while it strongly (albeit submaximally) suppressed *R. solani* (RGI and BCI 91 ± 1%) (Figure 2). Of all tested isolates, the *T. citrinoviridae* strains (NK 1/9, 127, and 11H1-4) showed the weakest activity against *R. solani*, with RGI values of 81 ± 2%, 78 ± 2%, and 85 ± 5% and BCI values of 84 ± 8%, 88 ± 6%, and 80 ± 3%, respectively. In summary, *S. sclerotiorum* was more susceptible to growth inhibition in dual culture confrontation by the *Trichoderma* isolates (RGI and BCI 100%) than *R. solani* (RGI between 78 and 100%; BCI between 80 and 100%). Overall, all *Trichoderma* isolates showed significant antagonistic activity against *S. sclerotiorum* and *R. solani*. The results obtained with the RGI and BCI methods were in good agreement, with complete overlap in the case of *T. harzianum*—1H1-27 and *R. solani*, where RGI and BCI were 91%, and slight discrepancies, as in the case of *T. citrinoviridae* strains NK 1/9, 127, and 11H1-4 against *R. solani*. The growth of *P. blakesleeanus* was maximally inhibited by all the *Trichoderma* spp. strains tested. The control cultures of *Trichoderma* spp. strains grown under the same conditions as the confrontation cultures with *P. blakesleeanus* are shown in Supplementary Material Figure S3. Images of the earlier stage of the confrontation dual culture plates, 3 days old, are shown in Supplementary Material Figure S2, and the control cultures of *Trichoderma* spp. strains grown under the same conditions as the confrontation cultures with *P. blakesleeanus* are shown in Figure S4. On the third day of dual culture, all *Trichoderma* spp. isolates already clearly inhibited the growth of *R. solani* and *P. blakesleeanus*, while *S. sclerotiorum* was inhibited by most strains. The apparently weak inhibition of *S. sclerotiorum* by strains 127, NK1/9, and 11H1-4 developed into an obvious inhibition on the fourth day of confrontation culture leading to a final strong inhibition after seven days of incubation.

### 3.3. Antifungal Potential of Trichoderma spp. Crude Extracts

**Figure 3:**
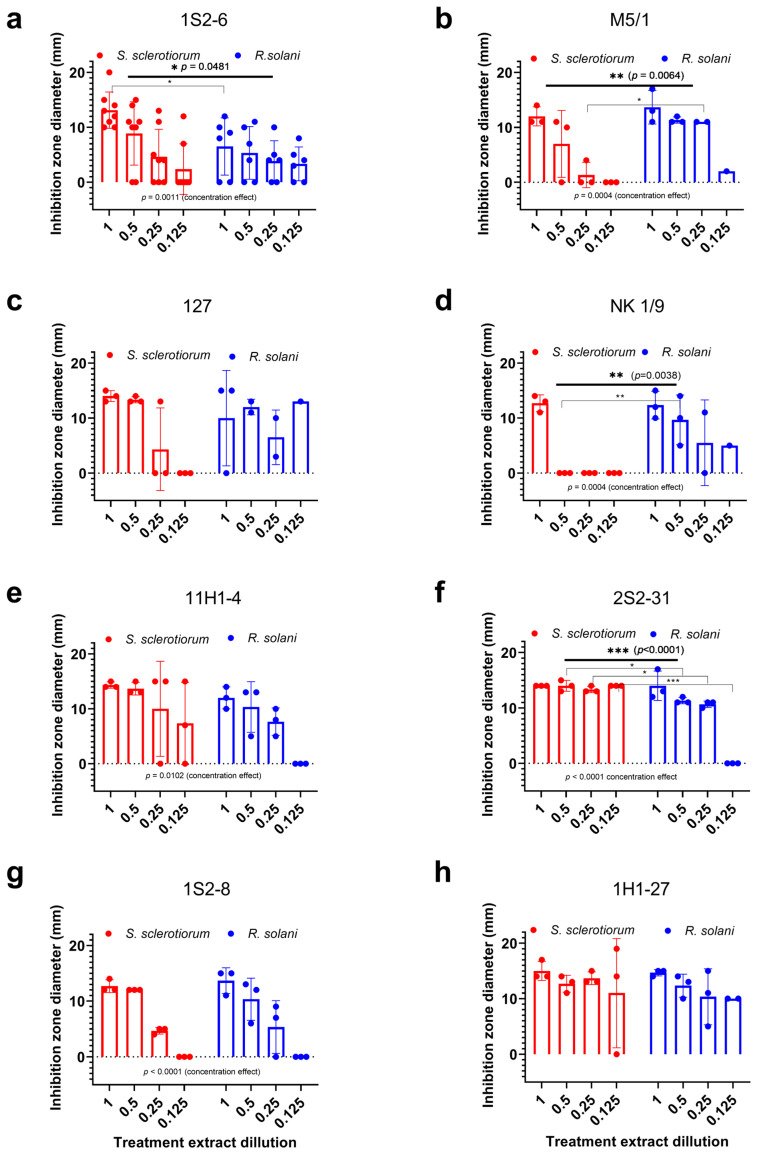
Concentration-dependent inhibition of *S. sclerotiorum* and *R. solani* growth by crude extracts of *Trichoderma* spp. (**a**) *T. harzianum*—1S2-6; (**b**) *T. citrinoviride*—M5/1; (**c**) *T. citrinoviride*—127; (**d**) *T. citrinoviride*—NK 1/9; (**e**) *T. citrinoviride*—11H1-4; (**f**) *T. longibrachiatum*—2S2-31; (**g**) *T. afroharzianum*—1S2-8; (**h**) *T. harzianum*—1H1-27. Diameter of the growth inhibition zones obtained in the well diffusion assay plotted against the corresponding extract dilution (1—undiluted; 0.5—50% extract; 0.25—25% extract; 0.125—12.5% extract). The bar charts show the mean ± SE; all points shown. Two-way ANOVA with the Holm–Sidac correction; the significance of the difference between the fungi is shown in the upper part of the graph, and the significance of the concentration dependence in the lower part. Significance levels: 0.05 (*), 0.01 (**), 0.005 (***).

The antagonistic effect of the crude chloroform extracts of *Trichoderma* spp. was tested on *S. sclerotiorum*, *R. solani*, and *P. blakesleeanus*. The diameters of the growth inhibition zones obtained by the well diffusion method plotted against the corresponding extract dilution (1—undiluted; 0.5—50% extract; 0.25—25% extract; 0.125—12.5% extract) are shown in Figure 3. Images of diffusion plates with wells are shown in Supplementary Figure S5. The crude extracts isolated from *T. harzianum*—1H1-27 and *T. afroharzianum* 1S2-8 showed a similarly strong inhibition of the growth of both phytopathogens. The *Trichoderma harzianum* strains had differential preference for significantly stronger inhibition of phytopathogens: *S. sclerotiorum* (M5/1, *p* = 0.0064) and *R. solani* (1S2-6, *p* = 0.0481). The *Trichoderma longibrachiatum*—2S2-31 extract significantly (*p* < 0.0001) inhibited the growth of *S. sclerotiorum* at all the tested concentrations, although it exerted strong inhibition of *R. solani* growth as well (defined as >75% of the maximum inhibition recorded for this fungal species in the presented assays). *Trichoderma citrinoviridae* strain 11H1-4 strongly inhibited the growth of both phytopathogens to a similar extent only at the highest concentrations tested, while strain NK 1/9 was a stronger antagonist (*p* = 0.0038) of *R. solani*, and strain 127 showed a similar but not statistically significant trend.

Next, we compared the response to the tested extracts in the well diffusion assay with the effect of alamethicin. Since the response to the tested extracts was dose-dependent and the maximum growth inhibition achieved, as well as the lowest dilution with a measurable effect, varied between the phytopathogens and *Trichoderma* strains, we first analyzed the sensitivity of the target fungi to alamethicin. Subsequently, we rated the potency of the extracts from the tested *Trichoderma* strains with respect to alamethicin.

**Figure 4:**
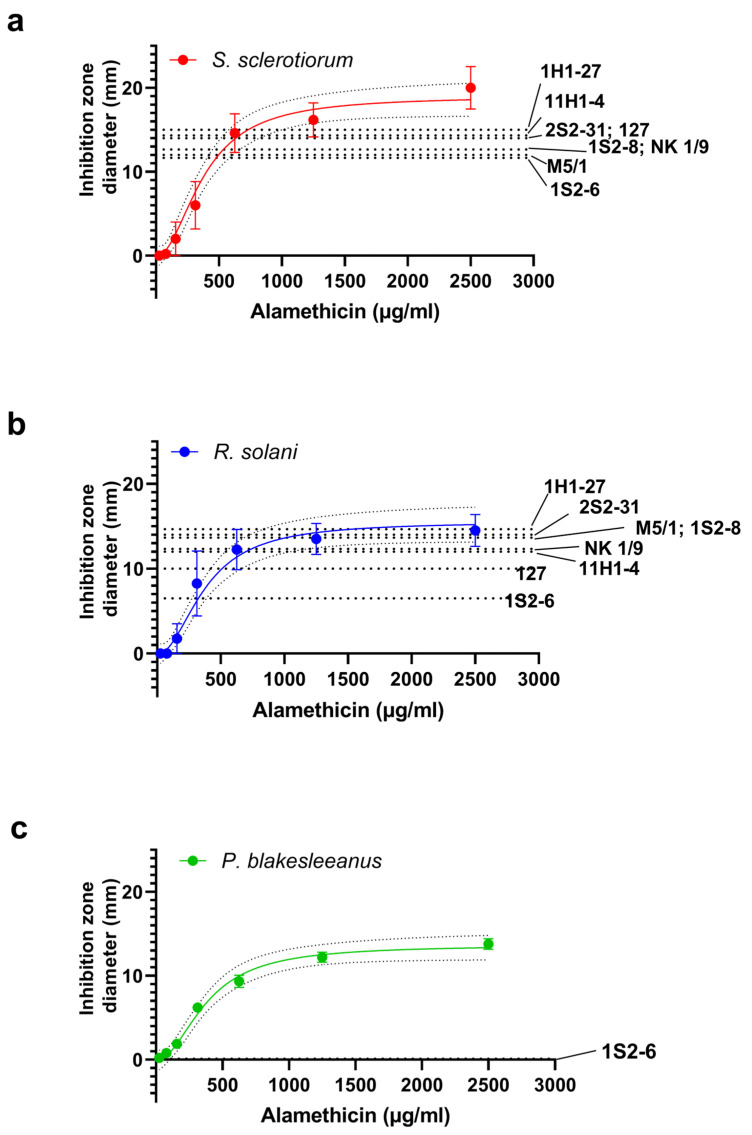
Sensitivity to growth inhibition by alamethicin and equivalence of the effects of undiluted extracts. Dose–response curves are constructed from the diameters of the inhibition zones in the well diffusion assay and fitted with a sigmoid curve. The confidence interval for the fit is shown by dotted lines. Mean ± SE, *n* = 4–9. On the right side, the corresponding extract codes of the *Trichoderma* strains are indicated for each value of d_max_ (dotted lines). (**a**) *S. sclerotiurum*; (**b**) *R. solani*; (**c**) *P. blakesleeanus*.

Alamethicin inhibited the growth of the fungi in a concentration-dependent manner (Figure 4), when the inhibition was expressed as the diameter of the growth inhibition zone. A sigmoid curve was fitted to the data with the following parameters: for *R. solani*, IC_50_ = 281 ± 61 µg/mL, n_h_ = 2.8 ± 1.6; for *S. sclerotiorum*, IC_50_ = 422 ± 88 µg/mL, n_h_ = 2.2 ± 0.9 (*n* = 3–8). From the IC_50_ values obtained, it appears that *R. solani* is more sensitive to alamethicin than *S. sclerotiorum*, although *S. sclerotiorum* forms apparently larger alamethicin-induced maximal inhibition zones (in mm): 20 ± 2.5 vs. 14.5 ± 1.9 for *R. solani*. Model likelihood testing by the extra sum-of-squares F test shows that both dose–response curves can be fitted with the same curve (*p* = 0.27), and the common parameters are IC_50_ = 360 ± 59 µg/mL, n_h_ = 2.3 ± 0.8, therefore, alamethicin sensitivity is not statistically significantly different between two studied phytopathogens. The alamethicin dose–response curve of *P. blakesleeanus* (Figure 4c) was found to be statistically different from the other two (*p* = 0.001), with the only difference in the lesser curve plateau value, while fit parameters IC_50_ and n_h_ are shared between all three curves.

The mean values of the diameters of the maximum inhibition zones determined for each crude extract tested are shown superimposed on the alamethicin dose–response curves as dotted lines in Figure 4. *Sclerotinia sclerotiorum* was inhibited by all extracts tested with similar mean d_max_ values (defined as the maximum mean diameter of the inhibition zone achieved with that combination of fungus and extract tested), in the region of the dose–response curve close to the maximum effect of alamethicin (Figure 4a). Remarkably, most extracts containing metabolites of the tested strains (named “Group I”; 11H1-4, NK 1/9, M5/1, S2-8, 2S2-31, 1H1-27) induce growth inhibition in *R. solani* that is comparable to the maximum inhibition achieved by alamethicin, as the d_max_ values obtained fall within the confidence interval for the plateau of the alamethicin dose–response curve (10.95 to 17.05 mm). The weakest inhibition was obtained by the 1S2-6 extract (d_max_ = 6.5 ± 2.1, *n* = 6), while the 127 extract produced inhibition close to the plateau value (Figure 4b). We conclude that *R. solani* is as sensitive to six group I extracts as to the commercial peptaibol alamethicin concentrate, somewhat in contrast to the incomplete growth inhibition in the direct confrontation experiment with some *Trichoderma* spp. strains of this group (1H1-27, 11H1-4 and NK 1/9). It should be mentioned that *T. citrinoviridae* strain 127 also produced incomplete growth inhibition of *R. solani* in direct confrontation, and since its extract had a submaximal inhibitory effect, this strain is less potent against *R. solani* both directly and through the action of released compounds. Strain 1S2-6 exerted relatively weak inhibition by the extract and strong inhibition (100% on all the fungi tested) by direct interaction in the confrontation experiment.

However, the growth inhibition of the *P. blakesleeanus* mycelium could not be effectively demonstrated by a selected tested crude extract from a *Trichoderma* spp. culture (*T. harzianum*—1S2-6) in the same experiment, by the agar plate well diffusion method, except at the highest concentration tested (Supplementary Figure S5). In order to prove that the test *Trichoderma*-derived extract inhibits *P. blakesleeanus* growth, an alternative method, bypassing the need for agar diffusion, was used. The growth of the submerged *P. blakesleeanus* culture was measured after a 24 h exposure to the extract of *T. harzianum* strain 1S2-6 at a concentration of 12.5%, which corresponds to the lowest concentration tested in the well diffusion assay (Figure 3).

### 3.4. Effects of T. harzianum-1S2-6 Extract on P. blakesleeanus Enzyme Activities

**Figure 5:**
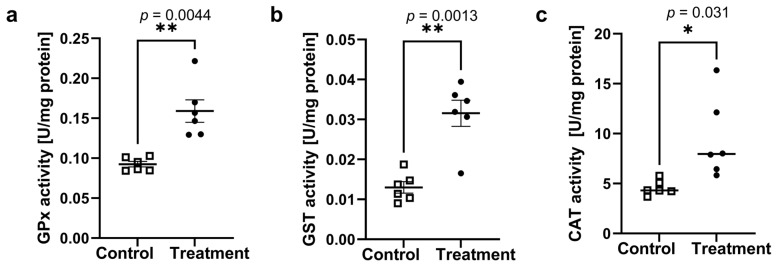
Effects of *T. harzianum*—1S2-6 peptaibol extracts on the specific activities of (**a**) glutathione peroxidase (GPx), (**b**) glutathione S-transferase (GST), and (**c**) catalase (CAT) in *P. blakesleeanus* mycelial lysates. Means and standard errors (SEs) were calculated from 3 biological replicates with 2 technical replicates each (*n* = 6). The horizontal line represents mean ± SE; all points shown. Significance levels: 0.05 (*), 0.01 (**).

*P. blakesleeanus* is a model fungus that has been shown to be tolerant to different stress conditions. It was here regarded as a suitable model for evaluating the biochemical response of fungi to stress imposed by peptaibol/*Trichoderma* extract treatment. The mycelial biomass after a 24 h incubation with the *T. harzianum* 1S2-6 extract was significantly lower (0.19 ± 0.06 g) (*p* = 0.031) than in the control group treated with vehicle (methanol) at the same concentration (0.44 ± 0.05 g). Thus, the growth inhibition by the 1S2-6 crude extract treatment in submerged culture demonstrates that *P. blakesleeanus* is susceptible to the antagonistic effect of the *T. harzianum* extract and that the growth inhibition of *P. blakesleeanus* by the *Trichoderma* isolates tested in the direct contact confrontation assay can be at least partially reproduced by the acellular chloroform crude extract. To assess the acute stress response of the *P. blakesleeanus* mycelium, the activity of the three defense-related enzymes was assayed, and the results are shown in Figure 5.

GPx activity increased significantly (*p* = 0.004), from 0.092 ± 0.008 to 0.16 ± 0.03 U/mg_protein_ (Figure 5a). The GST activity shown in Figure 5b also increased significantly (*p* = 0.001) from 1.3 ± 0.3 (×10^−2^) to 3.1 ± 0.8 (×10^−2^) U/mg_protein_. The catalase (CAT) activity increase, induced by treatment with the *T. harzianum*—1S2-6 extract (Figure 5c), was also significant (*p* = 0.03), from 4.6 ± 0.7 U/mg_protein_ under the control conditions to 9.4 ± 4 U/mg_protein_ in the treated mycelia. All the activity changes found are consistent with the stress induced by the crude extract, stimulating the oxidative defense response in the treated mycelia.

### 3.5. Confirmation of the Presence of Peptaibol-like Compounds in the Crude Extracts

**Figure 6:**
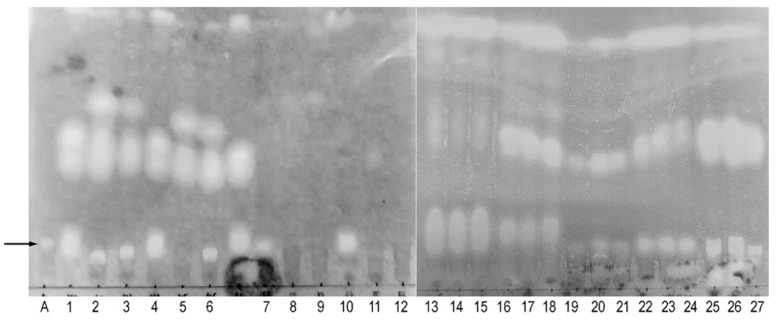
TLC of crude extracts stained for the presence of peptaibol-like compounds. Spots correspond to 4 µL alamethicin standard (A) or 8 µL of crude extracts (1–27). Crude extracts were obtained from *Trichoderma* monocultures (i.e., controls, lanes: 4–6, 10–12, 15, 18, 21, 24, 27) or from confrontation cultures of *Trichoderma* spp. with *R. solani* (1–3, 13, 16, 19, 22, 25) or *S. sclerotiorum* (7–9, 14, 17, 20, 23, 26). *Trichoderma* spp. strains are as follows: M5/1 (1, 4, 7, 10); 127 (2, 5, 8, 11); NK 1/9 (3, 6, 9, 12); 1H1-27 (13–15); 1S2-6 (16–18); 11H1-4 (19–21); 1S2-8 (22–24); 2S2-31 (25–27). Integral gel images are shown. The alamethicin position is indicated by the arrow on the left.

The crude extracts of all the *Trichoderma* spp. strains tested showed at least one peptaibol-like type band with low TLC mobility in the solvent system used, similar to the standard peptaibol alamethicin. The mobility of these alamethicin-like bands varied slightly between the strains, which could indicate a slightly different composition of the peptaibol-like compounds. Figure 6 shows that the tested *Trichoderma* spp. strains produce comparable amounts of alamethicin-like compounds under the control conditions (monoculture) and when confronted with one of the tested pathogens (dual culture), indicating that all investigated native *Trichoderma* spp. strains produce peptaibol-like secondary metabolites regardless of the immediate challenge by contact with phytopathogenic fungi. Most extracts of the *Trichoderma* spp. strains showed additional (one or two) peptaibol-like bands with higher mobility besides the alamethicin-like band. The nature of the dye used to visualize the bands (HCl hydrolysis followed by ninhydrin staining) suggests that all bands originate from peptide-based compounds. However, whether all three bands are de facto peptaibols and how many different types of peptaibol-like compounds exist remains to be confirmed, since TLC does not provide definitive confirmation of the chemical nature of compounds. Further analyses, such as HPLC or LC-MS/MS, would be necessary for the identification and structural characterization of peptaibols. Overall, we confirm the presence of peptaibol-like bands similar to those of standard alamethicin in the crude extracts, but their composition needs to be further explored.

The observed differences in the content of peptaibol-like compounds prompted us to calculate and compare the dry matter concentrations of the crude extract that induce a minimum observable inhibition of mycelial growth (MIMGI) of the tested phytopathogens (see Supplementary Table S2). For most extracts of the *Trichoderma* spp. strains, we found MIMGI < 5 mg/mL, with the exception of *T. afroharzianum*—1S2-8, which appeared to have a lower level of highly active antifungal compounds in the crude extract. *Trichoderma citrinoviridae*—11H1-4 and *T. longibrachiatum*—2S2-31 showed different MIMGI values for two phytopathogenic fungi, significantly higher for *R. solani* (*p* = 0.0193 and 0.0043, respectively), while the extract of *T. citrinoviridae*—NK 1/9 showed an inverse differential efficacy, with a significantly higher MIMGI for *S. sclerotiorum* (*p* < 0.0001). Overall, the MIMGI comparisons indicate that variations in the inhibitory effect of the crude extracts, as shown in Figure 3, are most likely caused by variations in the type and relative abundance of SMs contained in the extract and not by simple differences in the concentration of mycelial extracts obtained.

## 4. Discussion

Presented here are the results of measurements aimed at determining the degree of antagonistic activity of eight native *Trichoderma* isolates from Serbia against native isolates of the two common phytopathogenic fungi, *R. solani* and *S. sclerotiorum*, and a representative soil-borne fungus from the phylum Mucoromycota—*P. blakesleeanus*. All strains of *Trichoderma* spp. strongly inhibited the growth of phytopathogens (more than 75%), and some were 100% efficient in suppressing the growth of pathogens in vitro.

To allow comparison of the results obtained with the up-to-date literature overview, we used the two most common methods to quantify the results of the confrontation test: the RGI method and the BCI method. The BCI method was developed in 2006 [49] as a useful and reliable method based on image analysis, but it is more labor-intensive (requires photo documentation under strict conditions and image analysis), while the RGI method is still widely used [57,58]. The comparison of the data obtained with the two methods in our experiments showed a high degree of data similarity, although we cannot exclude the possibility that studies on other fungal species with a different geometry of colony growth could benefit from the application of BCI. The comparison of the results obtained with the results of a study that utilized native *Trichoderma* strains isolated from the same region indicates the higher antagonistic potential of our strains. Tančić et al. [57] evaluated the effect of *Trichoderma* isolates isolated from different soil types and localities in Vojvodina (Serbia) against *S. sclerotiorum* obtained from a sunflower crop in Rimski Šančevi (Vojvodina, Serbia) using a dual culture test. The result showed that the *Trichoderma* isolates exhibited radial growth inhibition against the phytopathogen (RGI between 36.1 and 52.2%). In the study by Zhang et al. [58], *T. harzianum* isolated from the rhizosphere of aloe (*Aloe arborescens*) in Zhoukou city (Henan province, China) was evaluated against *S. sclerotiorum* in the confrontation test, with an RGI of 56.3%. These studies showed a lower potential of the tested *Trichoderma* strains compared to the native *Trichoderma* strains originating from Serbia that were examined in our work (*S. sclerotiorum* RGI 100%). In the case of *R. solani*, *T. koningii* isolated from the rhizospheres of the Marathwada region of Maharashtra (India) inhibited mycelial growth by 67.7%, but other *Trichoderma* species tested showed mycelial growth inhibition of less than 50% [59]. Kotasthane et al. [60] investigated the efficacy of *Trichoderma* isolates from the culture collection of the Department of Plant Molecular Biology and Biotechnology, Raipur, Chhattisgarh, India, in suppressing mycelial growth of *R. solani* in vitro and obtained the following results: *T. virens* inhibited mycelial growth of *R. solani* by 90.10%, *T. viride* by 100.00%, *T. harzianum* by 79.56%, and *T. aureoviride* by 100.00%. Tamandegani et al. [50] evaluated the BCI values of *T. asperellum* and *T. longibrachiatum*, obtained from the Fungal Collection of Bu-Ali Sina University of Hamedan, Iran, against *R. solani* and obtained a BCI of 92.16% and 88.84%, respectively. In addition, Budha et al. [61] evaluated the BCI of *T. viride* and *T. harzianum* from the laboratory of the National Plant Pathology Research Center (NPPRC), Nepal Agricultural Research Council, Khumaltar, Lalitpur, Nepal, against *R. solani* and obtained results of 96.25% and 88.37%, respectively. In comparison to the results of our study, *T. harzianum* strains 1S2-6 and M5/1 and *T. longibrachiatum* strain 2S2-31 showed a BCI value of 100%. In another study by Singh et al. [62], *T. harzianum* isolated from soil (India) induced 99% growth inhibition of *R. solani* mycelia, which is similar to our results; the RGI of *T. harzianum*—1S2-6 and M5/1 against *R. solani* was 100%. It is worthwhile to note that in a dual culture test, commercial *T. harzianum* strain T22 inhibits phytopathogen *Fusarium culmorum*, with a maximal effect of slightly above 50%, after 14 days [63], while data on its in vitro activity against *R. solani* and *S. sclerotirum* are not available. In this study, a notable difference was found between the growth inhibitory effect of the same *Trichoderma* strain on different target species and the efficacy of different strains of the same *Trichoderma* species on different pathogens. This variability can influence the outcome of biocontrol measures and must be taken into account when selecting effective *Trichoderma* strains for designing consortia applicable in agriculture. Different *Trichoderma* species have been shown to have different gene expression patterns, reflecting their different strategies to achieve antagonism against fungi (expression of SMs, proteases and glucanases, cell wall hydrolytic enzymes or toxins) [64], which may be regulated by chromatin modification [65] and modulated by environmental factors [66]. It has already been shown that *Trichoderma* isolates from different geographical locations or ecological niches within the same species may exhibit different bioactivities [67]. Similar variability in the biocontrol effect of strains of the same *Trichoderma* species has been observed in several other studies [68], pointing out that among the variables that could be expected to affect fungi interactions during confrontation (such as temperature, pH, growth time), the biocontrol ability of the particular strain is the most important factor determining the outcome of confrontation with a phytopathogenic fungus [69].

To the best of our knowledge, the measurement of antagonism of *Trichoderma* spp. against *P. blakesleeanus* has not yet been described. *Phycomyces blakesleeanus* itself is not a plant pathogen, but a soil-borne, coprophilic fungus, which was used here as a well-studied fungus that can serve as a proxy for other species of the phylum Mucoromycota. The results obtained suggest that *Trichoderma*-based biocontrol consortia containing the strains tested here may be efficient against phytopathogenic members of the phylum Mucoromycota. Based on the fact that *P. blakesleeanus* has all the characteristics of Mucoromycota that could make them less susceptible to *Trichoderma* spp., such as specificity of cell wall composition and very rapid growth, we propose that *P. blakesleeanus*, as a well-characterized model organism, could be a first approximation for modeling the interaction between Mucoromycota and *Trichoderma* fungi. In particular, the very fast growth of *P. blakesleeanus* might be expected to provide some protection against the antagonistic mechanisms of *Trichoderma* competition and overgrowth [7]. Our data from the early stages of dual cultures, as shown in Figure S2, refute this assumption and show that *P. blakesleeanus* is effectively inhibited by all the *Trichoderma* strains tested. However, these conclusions still need to be further tested on other agriculturally or medically relevant Mucoromycota fungi. All *Trichoderma* strains showed maximum inhibition in the complete confrontation test against *P. blakesleeanus* (100% RGI and BCI). This is in agreement with the work by Purwantisari et al. [70], which showed that *T. harzianum* is a moderately effective inhibitor of the growth of *Mucor circinelloides*. This study contributes to the understanding of the dependence of *Trichoderma* antagonism on the specific features of the cell wall composition of filamentous fungi, as the chitosan-rich cell wall of fungi from the phylum Mucormycota has a very different architecture from the chitin- and beta-glucan-rich cell wall of fungi from the phyla Ascomycota and Basidiomycota [18].

In general, the *Trichoderma* isolates extracts tested had high antagonistic activity against the severe phytopathogens *S. sclerotiorum* and *R. solani* and against *P. blakesleeanus*. The antifungal activity of the crude extracts of the *Trichoderma* isolates against the investigated phytopathogens proved to be dependent on the target strain in some cases. For example, the extract obtained from *T. harzianum* strain M5/1 was more effective against *S. sclerotiorum*, while the extract from strain 1S2-6 of the same species was more effective against *R. solani*. Based on the comparison of the inhibitory effect of a particular strain in direct interaction with its chloroform crude extract, it is clear that some strains appeared to exert their inhibitory effect mainly by direct contact or by releasing compounds not present in the chloroform extracts, while others could achieve maximum inhibition through the action of small inhibitory molecules present in the chloroform crude extracts. We compared the effect of the crude extracts with that of the peptaibol standard alamethicin and found that the extracts of Group I strains are growth inhibitors that are as potent as alamethicin, while the effect of some extracts depends on the pathogen to be inhibited, suggesting that the same extract can achieve different levels of inhibitory effects on different fungi. Under our experimental conditions in the well diffusion test, alamethicin inhibited the growth of all three fungi representatives tested, with a similar IC_50_, which was below 400 µg/mL, while the minimum concentration that had an inhibitory effect was 150 µg/mL. Recently, a test of alamethicin on artificial lipid bilayers confirmed that its activity is modulated by changes in the material properties of the lipid bilayer, as well as curvature and elasticity [71]. It is noteworthy that the efficacy of alamethicin against all three tested fungi from the phyla Basidiomycota, Ascomycota, and Mucoromycota is essentially the same, according to our data, and the dose–response curves overlap, indicating that the outcome of its activity is not influenced by possible differences in the underlying membrane properties between the target fungi. Although alamethicin is considered the best-investigated peptaibol agent for which structural and membrane activity models have been developed [72,73], to our knowledge, the exact inhibitory potency against individual phytopathogenic fungi has not been recently scrutinized [74]. It is known that alamethicin is a potent membrane-active bactericidal agent with an MIC in the low µM range (up to 12 µM) against *Mycoplasma* and *Spiroplasma* spp. [75]. Other pure peptaibols isolated from *T. asperellum* had an MIC of 800 µg/mL or less against *Colletotrichum gloeosporioides*, *Botrytiscinerea* sp., *Alternaria alternata*, and *Fusarium oxysporum* in different assays mixed with PDA on whole plates [76]. Since Mueller et al. [77] measured the IC_50_ for growth inhibition of *S. sclerotiorum* by the chemical fungicides vinclozolin and thiophanate-methyl to be 0.6 and 2.2 µg/mL [77], our data confirm that pure peptaibols alone must be used as chemical growth inhibitors for *S. sclerotiorum* at a concentration several orders of magnitude higher. The numerous advantages of using *Trichoderma*-based biocontrol agents are linked to the synergistic actions of all elements of the *Trichoderma* antifungal repertoire [78], as well as to the sustainability of biological treatments of crops as opposed to chemical treatments [79].

A recent study by Tamandegani et al. [50] showed that a crude extract containing peptaibols released by *Trichoderma* inhibited the germination of the conidia of the tested phytopathogenic fungi (*Fusarium* sp., *Alternaria solani*, and *R. solani*). In other studies, the effects of crude chloroform-containing peptaibol extracts of *T. gamsii* and *T. koningiopsis* [80] on the inhibition of mycelial growth of *A. alternata* and *R. solani* were demonstrated. In addition, the antagonistic effect of peptaibol trichokonins extracted and purified from *T. koningii* was demonstrated against *R. solani* [81]. Isolated peptaibols or crude extracts containing peptaibol from *Trichoderma* spp. have been shown to exhibit antifungal activity against a broad spectrum of phytopathogens [15].

Furthermore, we have shown that *P. blakesleeanus*, a member of the phylum Mucoromycota, shows a sensitivity to alamethicin similar to that of *R. solani* and *S. sclerotiorum*. The significantly smaller maximum radii of the inhibition zones compared to the tested phytopathogens, which are reflected in the lower value of the plateau of the dose–response curve to alamethicin obtained for *P. blakesleeanus*, are reminiscent of the non-measurable diameters of the growth inhibition zones of *P. blakesleeanus* that we obtained in the *Trichiderma* spp. extract testing using well diffusion plates. Since the same extract, even at low concentration, caused substantial growth inhibition of *P. blakesleeanus* and a concomitant increase in oxidative defense enzymes, it can be safely assumed that the agar plate well diffusion test is not a suitable method to assess the sensitivity of *P. blakesleeanus* to peptaibol-containing agents. The results of such tests could falsely indicate low efficacy of peptaibols and peptaibol-containing extracts of *Trichoderma* in inhibiting the growth of *P. blakesleeanus.*

There are numerous reports describing that the interaction with *Trichoderma* species causes an increase in the activities of antioxidant enzymes in plants [82,83]. Some *Trichoderma* strains induce a sustained increase in the expression of CAT and other antioxidant defense enzymes as part of the complex interactions with plants that lead to protective and growth-promoting effects [84]. It appears that *Trichoderma* exerts the described effects not only by stimulating ROS production by the plant but also by producing ROS itself [85]. The effects of *Trichoderma* on the antioxidant metabolism of plants have been well studied, but it seems that these effects during interfungal interactions have received little attention, and there are few data on the effects of *Trichoderma* spp. on the antioxidant enzyme activities of other fungi. During antagonistic interactions between *Trichoderma* and other fungi, similar ROS production patterns were observed in confronted fungi as those reported in plants interacting with *Trichoderma*. For example, the role of ROS in the mycoparasitic interaction between *T. koningii* and *Phallus rubrovolvatus* was investigated, and increased levels of ROS were detected around the infection sites, leading to oxidative damage in the host fungus [86]. *Sclerotinia sclerotiorum* exposed to volatile compounds produced by *Trichoderma* was found to have reduced mycelial growth and sclerotia production, as well as upregulated GST gene expression and elevated GST enzyme activity [87]. Non-volatile compounds (SM and H_2_O_2_) produced by *Trichoderma* spp. also led to severe oxidative stress [88]. A number of enzymes, including SOD and peroxidase/catalase, were upregulated in *Fusarium culmorum* mycelia after treatment with *Trichoderma* extracts [89]. *Phallus rubrovolvatus* showed significant ROS production and an increase in redox-active defense enzymes (superoxide dismutase, Mn peroxidase, phenylalanine ammonia-lyase, polyphenol oxidase, and laccase) after infection with *T. koningii* [86]. We demonstrated that the exposure of *P. blakesleeanus* mycelia to *Trichoderma* extract resulted in a significant increase in the activities of the defense enzymes CAT, GPx, and GST. Antioxidant enzymes play a crucial role in maintaining cellular redox balance, which is vital for mycelial survival in selenite-treated *P. blakesleeanus*, as evidenced by increased activities of CAT, GST, GPx, and glutathione reductase [90]. Increased enzyme activities indicate that the crude extract of *Trichoderma* induced significant oxidative stress in the treated *P. blakesleeanus* mycelia. The catalase activity of mycelia has been shown to increase under various stress situations, including pathogen infection, to maintain redox balance, resulting in resistance to environmental stress [91]. Intensive H_2_O_2_ production was detected in fungal cells attacked by *Trichoderma* spp. or treated with its extracts [86,92], but there is also evidence that *Trichoderma* can release substantial amounts of H_2_O_2_ to drive mycoparasitism [88]. The increased CAT activity in *P. blakesleeanus* mycelia observed in this work can be interpreted as a response to the increased H_2_O_2_ production induced by the *Trichoderma* SM contained in the extracts. GPx and GST are enzymes involved in the detoxification of various peroxides and hydroperoxides, as well as xenobiotics [93,94]. The observed increase in the activities of these enzymes is consistent with acute stress in the mycelia treated with *Trichoderma* extracts, which is probably due to both overproduction of ROS and the need for detoxification of *Trichoderma* SMs. The increase in GPx and GST activities reflects the ability of the fungus to counteract oxidative stress, especially in the exponential growth phase [95]. However, the effectiveness of these enzymes in mitigating damage depends on the severity of the attack, which is likely to be influenced by the *Trichoderma* species and strain involved.

We have shown that the induction of the antioxidative system in host fungi can be induced by acellular chloroform extract, suggesting that the compounds present in the crude extract may exert a synergistic action with the ROS produced by *Trichoderma* species during mycoparasitism and the plant’s own defenses against phytopathogenic fungi. The results obtained can be explained as a consequence of the disruption of the cell wall and plasma membrane of the target fungi by the fungicidal components of the extract, as shown in the studies on the fungicidal activity of other *Trichoderma*-derived SMs [96]. The presence of peptaibols among the SMs in the crude extracts was confirmed by TLC, which is considered a useful method for preliminary screening, separation, and identification of peptide compounds [97]. TLC of crude chloroform extracts has previously been used for the screening and separation of peptaibols in *Trichoderma* extracts [98], and the patterns of peptaibol-like compounds produced by individual *Trichoderma* strains revealed by TLC appear to vary depending on several factors, although interaction with pathogens in dual cultures does not appear to alter the TLC profile of crude extracts. However, detailed studies focusing on the identification of peptaibol species upon an interaction between *Trichoderma* and target fungi showed an increase in the total amount of peptaibols as well as some differences in the peptaibol species and their relative amounts produced when confronted with a range of phytopathogens, including *R. solani* [50], or with acellular fragments of phytopathogens acting as elicitors [76]. The TLC profiles of the crude extracts described here should probably be interpreted with caution, as they demonstrate the presence of several different types of peptide molecules with peptaibol-like properties. All strains showed one band with low TLC mobility, similar to alamethicin. Strains 127 and NK1/9 showed two more bands, while all remaining strains showed one additional band. The chemical nature of all these bands should be confirmed by more sophisticated structural analyses.

The differences in the TLC profiles, as well as the differences in the inhibitory activity of the extracts from different strains, suggest that different strains, even when grown under exactly the same conditions, may produce different peptaibol-like active compounds and other SMs, even when strains of the same species are compared. The observed differences in the content and efficacy of extracts containing small peptide compounds, including peptaibols, should be further explored with the aim of improving existing formulations and developing new formulations of bioactive control agents (BCAs) based on *Trichoderma* species. Since the application of biocontrol agents to specific plants or soils may yield results that differ from the effects obtained in vitro, further experiments in vivo will be important for fully determining the potential of the tested native *Trichoderma* strains as BCAs.

## 5. Conclusions

There is a need for more environmentally friendly solutions for combating phytopathogenic fungi in agricultural practice. In the present paper, eight *Trichoderma* native isolates were established as potent inhibitors of phytopathogens *R. solani* and *S. sclerotiorum*, as well as *P. blakesleeanus*, as a representative of Mucoromycota. Both biological fungicide variants, *Trichoderma* isolates and their chloroform extracts, show strong antifungal activity, which can be at least partly attributed to their peptaibol-like compounds. The *Trichoderma* strains described here are a promising starting point for the development of new biocontrol consortia for agricultural applications.

## Figures and Tables

**Table 1:** Accession numbers of the identified *Trichoderma* spp. and phytopathogens.

Strain Code	Species	Accession Number
1S2-8	*Trichoderma afroharzianum*	PQ496494
11H1-4	*Trichoderma citrinoviride*	PQ496497
1H1-27	*Trichoderma harzianum*	PQ496510
1S2-6	*Trichoderma harzianum*	PQ496529
2S2-31	*Trichoderma longibrachiatum*	PQ496530
127	*Trichoderma citrinoviride*	PQ496531
NK 1/9	*Trichoderma citrinoviride*	PQ601324
M5/1	*Trichoderma harzianum*	PQ651500
K-500	*Sclerotinia sclerotiorum*	PQ496532
K-499	*Rhizoctonia solani*	PQ496533

## Data Availability

The original contributions presented in this study are included in the article/Supplementary Material. Further inquiries can be directed to the corresponding authors.

## Supplementary Materials

The following supporting information can be downloaded at: https://www.mdpi.com/article/10.3390/jof11070535/s1, Table S1. Primers used for amplification and their corresponding PCR profiles. Figure S1. Phylogenetic tree of *Trichoderma* isolates and reference strains based on ITS and *tef1* region sequences. Figure S2. Representative images of Petri plates showing the results of confrontation after 3 days incubaton. *Trichoderma* spp. strains with each target fungi tested. Figure S3. Representative images of the control *Trichoderma* spp. cultures grown in *P. blakesleeanus* growth conditions for 7 days. Figure S4. Representative images of the control *Trichoderma* spp. cultures grown in *P. blakesleeanus* growth conditions for 3 days. Figure S5. Representative images of a well diffusion essay. Petri plates showing the antifungal effect of commercial peptaibol alamethicin and crude chloroform extracts from *Trichoderma* spp. on the growth of *S. sclerotiorum*, *R. solani* and *P. blakesleeanus*. Table S2. Minimal concentrations of crude extract dry matter that induce observable growth inhibition (MIMGI) of *R. solani* and *S. Sclerotiorum*. Figure S6. Agar well diffusion essay data showing concentration-dependent inhibition of *S. sclerotiorum* and *R. solani* growth by crude extracts of *Trichoderma* spp.
