# Supplementary material for: Biocontrol Potential of Native Trichoderma Strains Toward Soil-Borne Phytopathogenic and Saprotrophic Fungi
Source: J Fungi (Basel). 2025 Jul 18;11(7):535. doi: 10.3390/jof11070535 (PMC12295951; doi:10.3390/jof11070535)
Supplement: Supplementary file 1 [file jof-11-00535-s001.zip › jof-3695971-supplementary.pdf]

Article

Biocontrol potential of native *Trichoderma* strains toward soil-borne phytopathogenic and saprotrophic fungi

Supplementary Material

Table S1. Primers used for amplification and their corresponding PCR profiles

Primers name	Sequence (5'-3')	Gene
**ITS1-F**	TCCGTAGGTGAACCTGCGG	**ITS1 and ITS2 regions, separated by the 5.8S gene**
**ITS4-R**	TCCTCCGCTTATTGATATGC	
**EF1-728F**	CATCGAGAAGTTCGA-GAAGG	**Translation elongation factor 1 α (*tef1*) gene**
**TEF1LLErevR**	AACTTGCAGGCAATGTG	

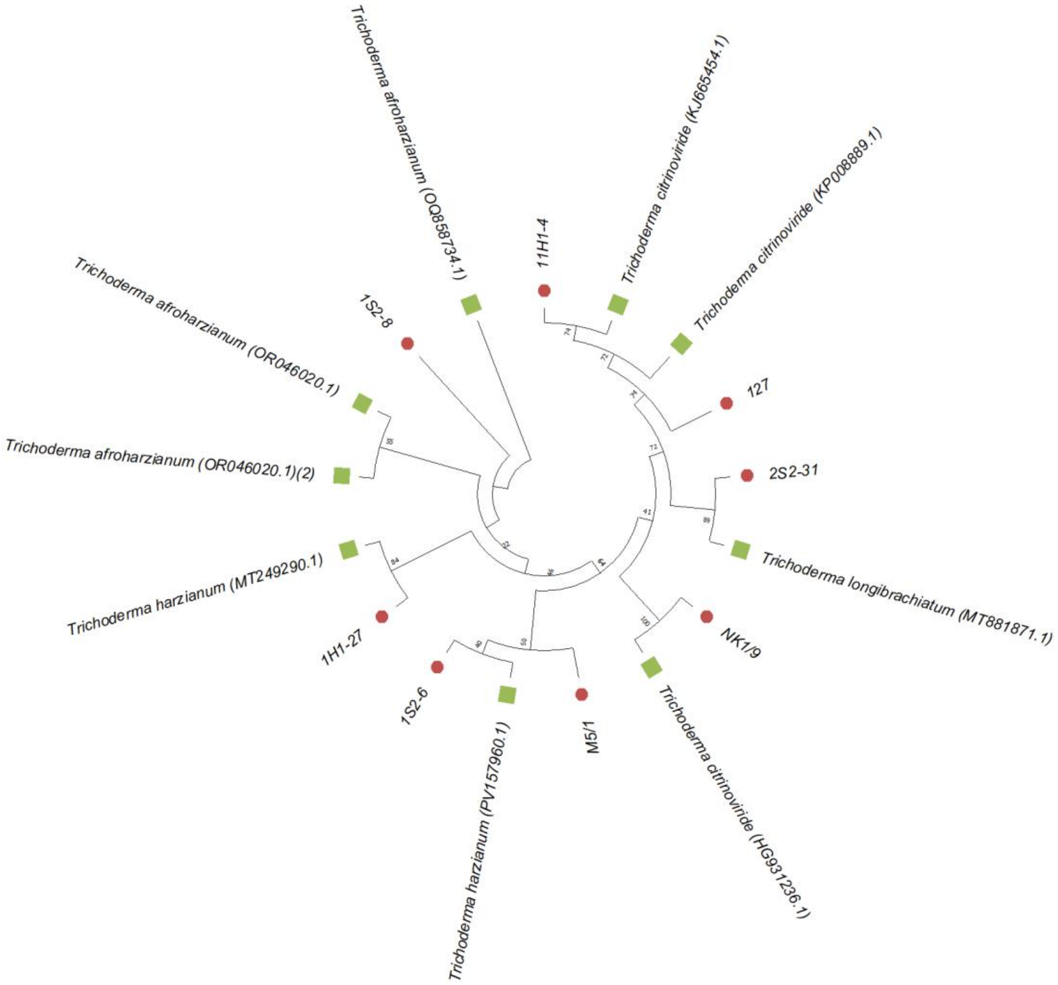

Figure S1. Phylogenetic tree of *Trichoderma* isolates and reference strains based on ITS and *tef1* region sequences. The circular phylogenetic tree was constructed to illustrate the evolutionary relationships between the selected *Trichoderma* isolates (marked with red circles) and selected reference strains retrieved from GenBank (marked with green squares). Sequences were aligned using ClustalW, and the tree was generated using the Neighbor Joining algorithm with bootstrap support values indicated at the nodes.

Method for generating phylogenetic tree

The phylogenetic tree was constructed by the neighbor-joining method and the distances were calculated with the Kimura two-parameter model. The percentage of replicate trees in which the associated taxa clustered together in the bootstrap test (1000 replicates) is shown next to the branches. The tree is drawn to scale, with branch lengths in the same units as those of the evolutionary distances used to infer the phylogenetic tree. The analysis involved 4 nucleotide sequences. All positions with less than 95% site coverage were eliminated. That is, fewer than 5% alignment gaps, missing data, and ambiguous bases were allowed at any position.

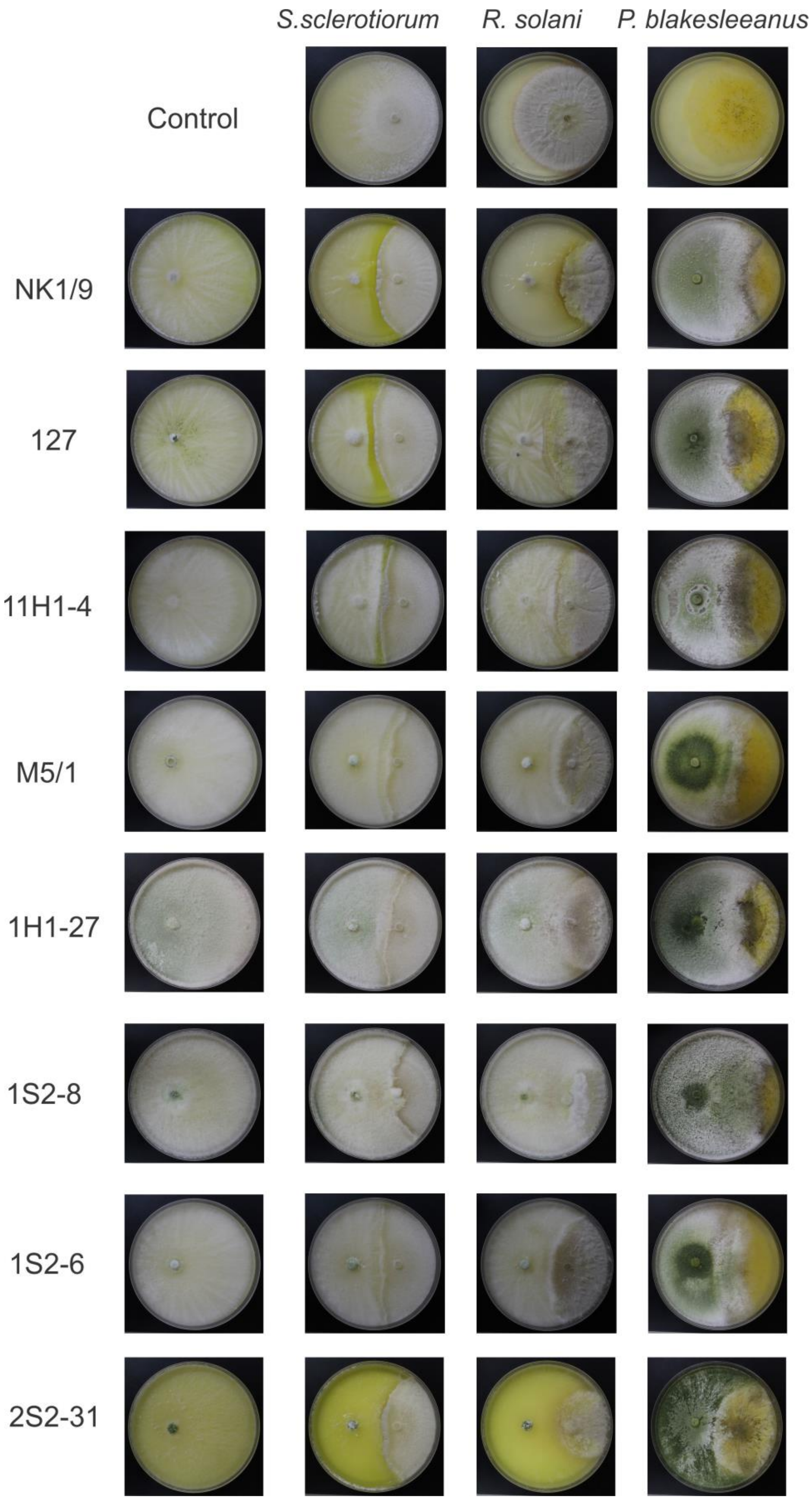

Figure S2. Representative images of Petri plates showing the results of confrontation after 3 days incubation. *Trichoderma* spp. strains with each target fungi tested. From top to bottom, strains of *Trichoderma* spp.: NK 1/9, 127, 11H1-4, M5/1, 11H1-27, 1S2-8, 1S2-6, 2S2-31. All experiments were performed triplicate. The images on the far left show *Trichoderma* spp. control cultures obtained in phyto-pathogen growth conditions. Control cultures corresponding to *P. blakesleeanus* confrontation plates are shown in Figure S4.

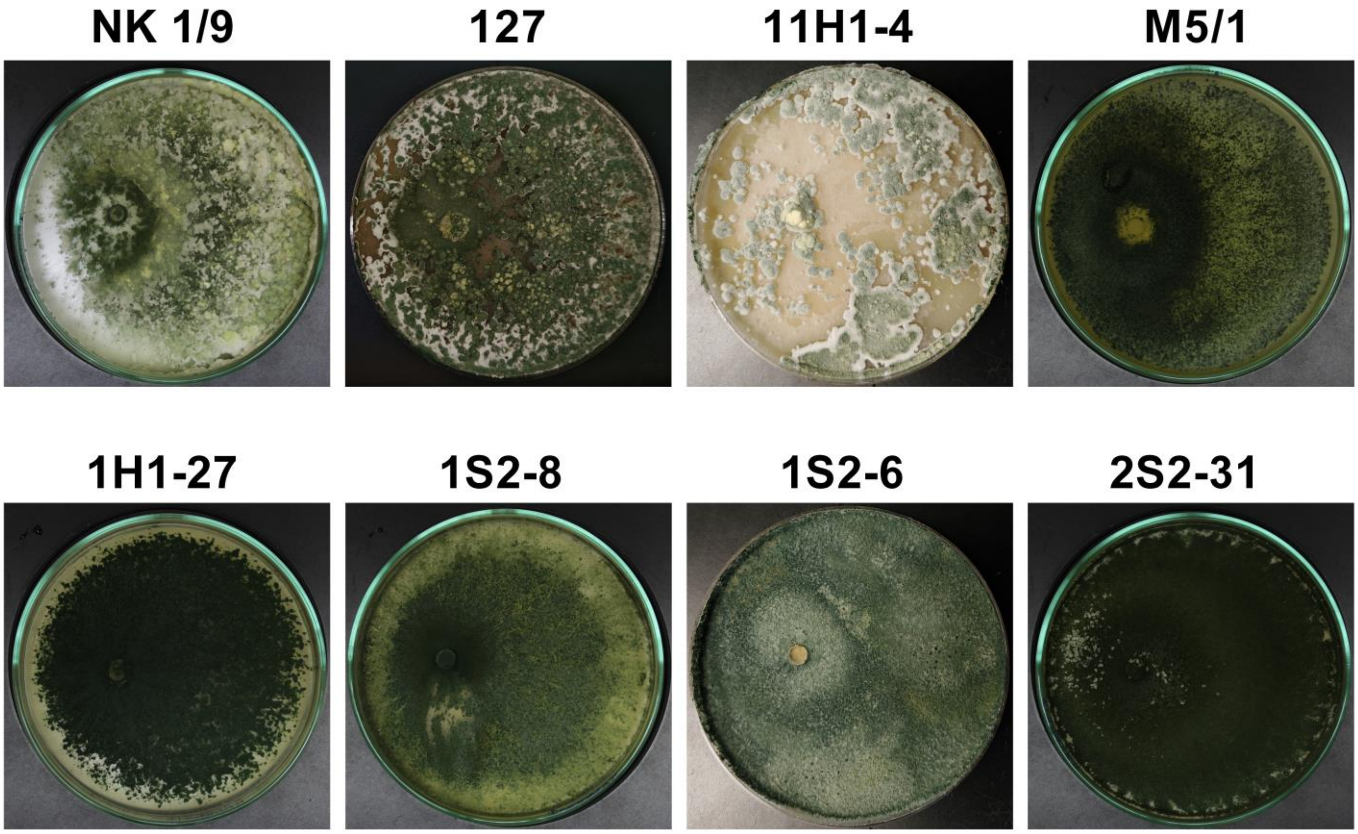

Figure S3. Representative images of the control *Trichoderma* spp. cultures grown in *P. blakesleeanus* growth conditions for 7 days. From left to right, *Trichoderma* ssp. strains: NK 1/9, 127, 11H1-4, M5/1, 11H1-27, 1S2-8, 1S2-6, 2S2-31. All experiments were done in triplicate. The diameter of the Petri plates: 90 mm.

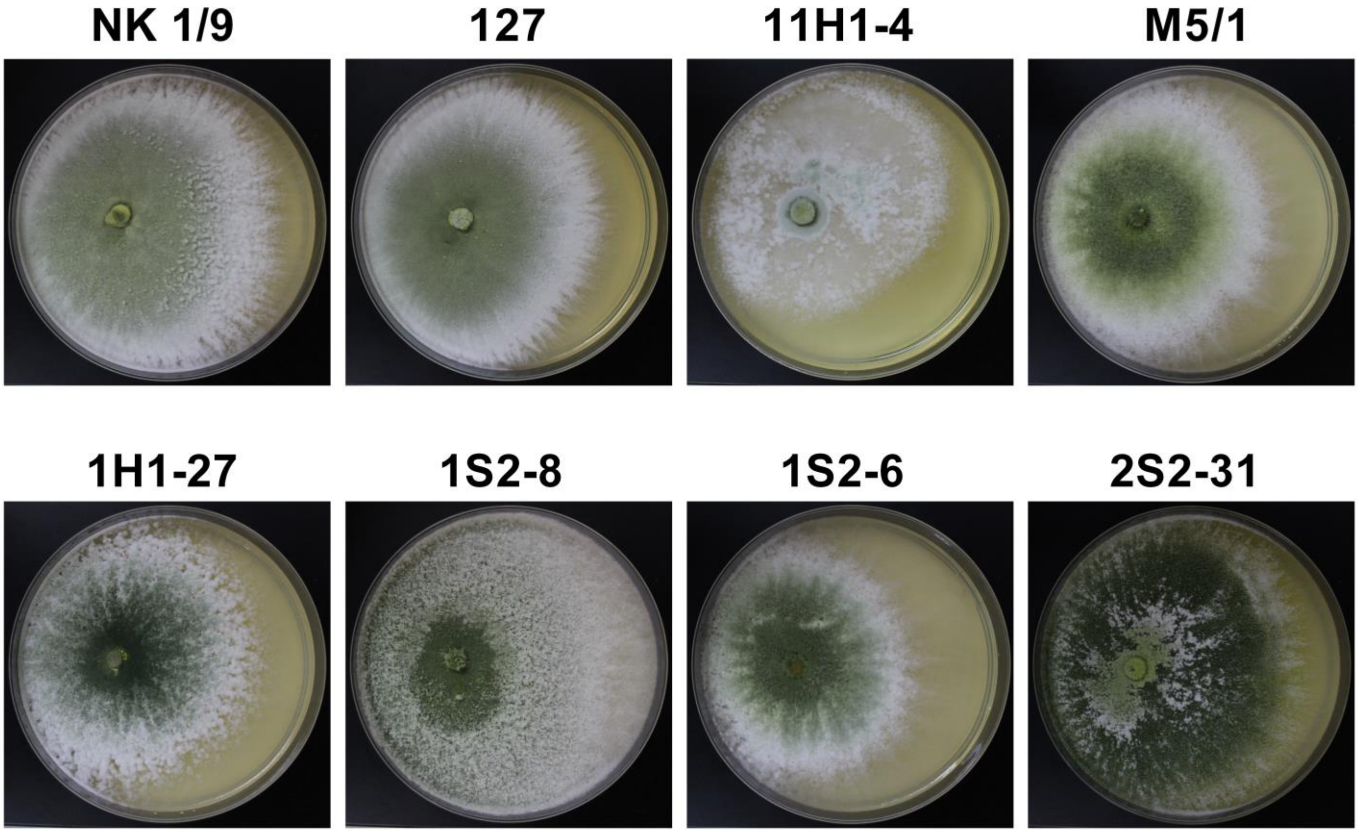

Figure S4. Representative images of the control *Trichoderma* spp. cultures grown in *P. blakesleeanus* growth conditions for 3 days. From left to right, *Trichoderma* ssp. strains: NK 1/9, 127, 11H1-4, M5/1, 11H1-27, 1S2-8, 1S2-6, 2S2-31. All experiments were done in triplicate. The diameter of the Petri plates: 90 mm.

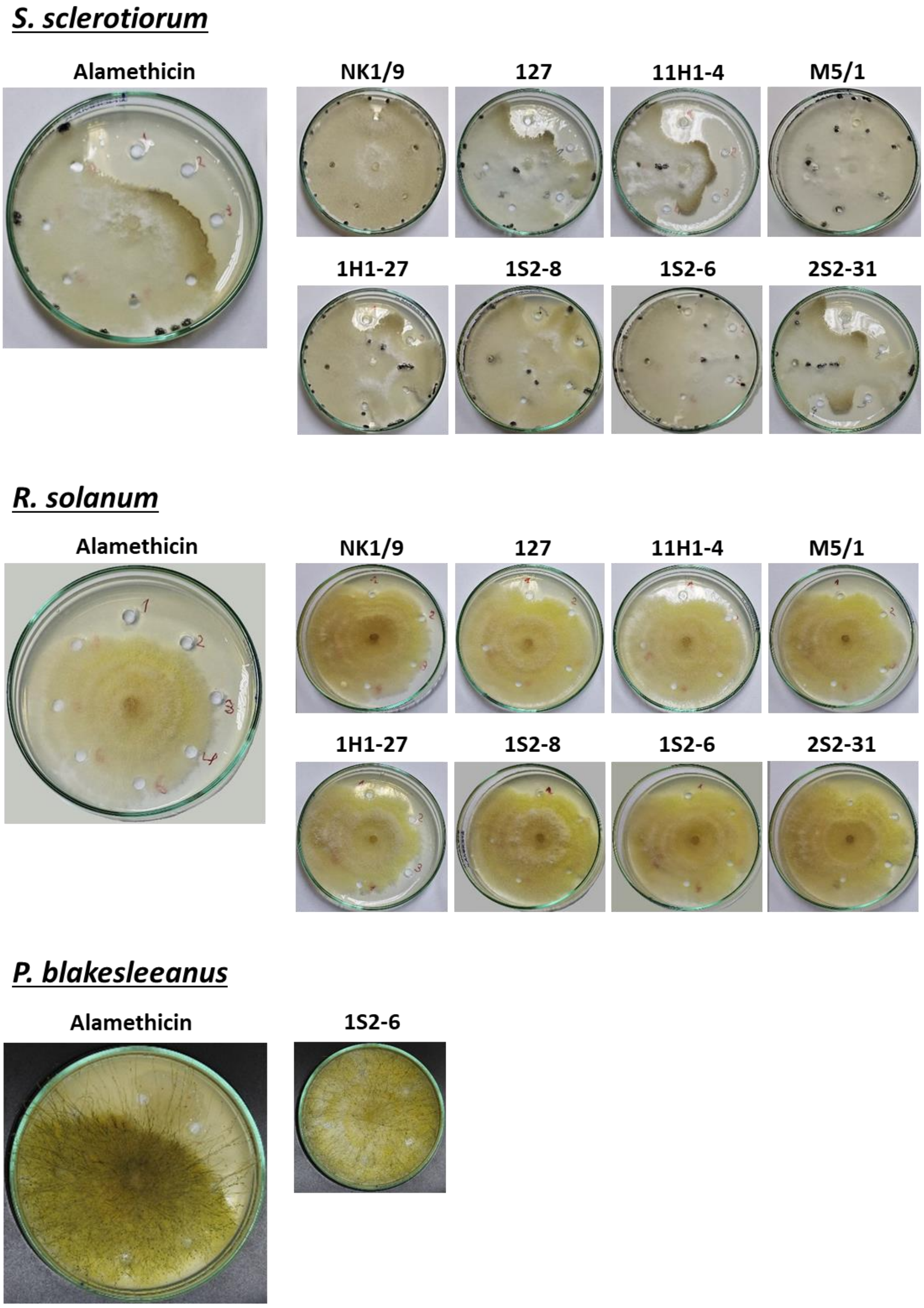

Figure S5. Representative images of a well diffusion essay. Petri plates showing the antifungal effect of commercial peptaibol alamethicin and crude chloroform extracts from *Trichoderma* spp. on the growth of *S. sclerotiorum, R. solani* and *P. blakesleeanus*. For the alamethicin microdilution testing, eight equidistant round wells of 5 mm in diameter were bored into agar plates on periphery of Petri dish and 25 μL of alamethicin was added in sequential dilutions (2500 μg/mL, 1250 μg/mL, 625 μg/mL, 312.50 μg/mL, 156.25 μg/mL, 78.25 μg/mL and 25 μg/mL) into the wells (labelled with the numbers 1 to 7 on the underside of the Petri dish). The control well was filled with same volume of methanol (number 8). For the crude extracts of *Trichoderma* spp., five round wells with a diameter of 5 mm were bored into agar plates on the periphery of Petri dish, equally distant from the centre of the plate, and filled with 25 μL of the serial dilution of the crude peptaibol extract in methanol (100%, 50%, 25%, 12.5%) (labelled with the numbers 1 to 4). Methanol was used in one well as a control (number 5). All experiments were done in triplicate. The diameter of the Petri plates: 90 mm.

Table S2. Minimal concentrations of crude extract dry matter that induces observable growth inhibition (MIMGI) of *R.solani* and *S. Sclerotiorum*

Trichoderma spp. strain	*R. solani* MIMGI mean (mg/mL)	SD	n	*S. sclerotiorum* MIMGI mean (mg/mL)	SD	n
1S2-6	1.86[a]	0.08	3	3.88[a]	2.11	3
M5/1	1.1[a]	1.04	3	4.57[a]	2.03	3
127	0.52[a]	0.31	3	2.16[a]	0.2	3
NK 1/9	0.58[a]	0.49	3	9.74[b]	3.22	3
11H1-4	6.05[b]	1.79	3	1.45[a]	0.43	3
2S2-31	7.06[b]	2.4	3	1.69[a]	0.58	3
1S2-8	6.49[b]	0.57	3	6.49[a]	3.81	3
1H1-27	2.84[a]	1.46	3	2.84[a]	0	3

One-way ANOVA testing of MIMGI obtained for *R. solani* showed that extracts originating from *T. citrinoviridae* – NK 1/9, *T. longibrachiatum* - 2S2-31 and *T. afroharzianum* - 1S2-8 significantly differed in minimal concentration required to elicit inhibitory effect from the other tested strains extracts ($p < 0.0001$), displaying reduced inhibitory potential of secreted SM in chloroform extracts on *R. solani*, compared to other tested strains (indicated with b). Same type of analysis of MGMI data obtained for *S. sclerotiorum* showed that only *T. citrinoviridae* – NK 1/9 extract was significantly different (<0.05) from other strain extracts in their potency to inhibit growth of *S. sclerotiorum* (indicated by b).

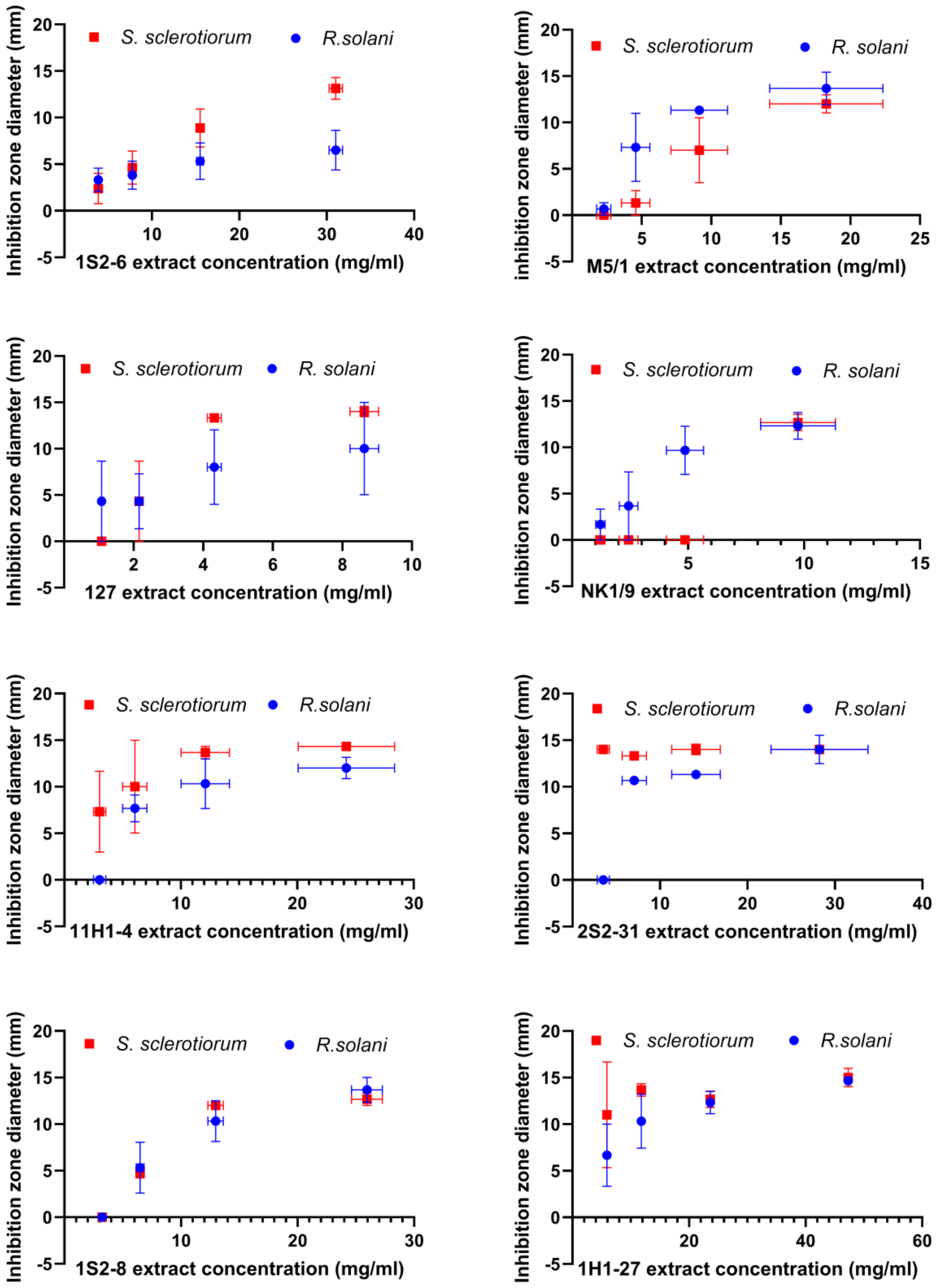

Figure S6. Agar well diffusion essay data showing concentration-dependent inhibition of *S. sclerotiorum* and *R. solani* growth by crude extracts of *Trichoderma* spp. : *T. harzianum* - 1S2-6; *T. harzianum* - M5/1; *T. citrinoviride* - 127; *T. citrinoviride* - NK 1/9; *T. citrinoviride* - 11H1-4; *T. longibrachiatum* - 2S2-31; *T. afroharzianum* - 1S2-8; *T. harzianum* -1H1-27. Diameter of the growth inhibition zones plotted against the corresponding concentration of extract dry mass. Mean ± SE, *n*=3-8. The X axis errors are calculated by multiplying each concentration by corresponding relative error for that extract. Calculated from the data shown in Figure 3.
